# Diagnostic Methods for the Prenatal Detection of Cleft Lip and Palate: A Systematic Review

**DOI:** 10.3390/jcm13072090

**Published:** 2024-04-03

**Authors:** Ana Baeza-Pagador, Ana Tejero-Martínez, Lucas Salom-Alonso, Sara Camañes-Gonzalvo, Verónica García-Sanz, Vanessa Paredes-Gallardo

**Affiliations:** 1 Orthodontics Teaching Unit, Department of Stomatology, Faculty of Medicine and Dentistry, University of Valencia, 46010 Valencia, Spain; abapa2@alumni.uv.es (A.B.-P.); anatejeromtz@gmail.com (A.T.-M.); sara.camanes@uv.es (S.C.-G.); veronica.garcia-sanz@uv.es (V.G.-S.); 2 Department of Maxillofacial Surgery, La Fe Hospital, 46026 Valencia, Spain; salom_luc@gva.es

**Keywords:** cleft lip, cleft palate, fetus, pregnancy, 2D ultrasound, 3D ultrasound, MRI, diagnosis, specificity, sensitivity

## Abstract

**Background:** Accurate prenatal diagnosis of cleft lip and palate is essential to discuss severity prediction, perform appropriate parental counseling, and, at last, establish long-term treatment planning. The aim of this systematic review was to analyze the accuracy of various imaging techniques for the prenatal diagnosis of cleft lip and palate, assess the pregnancy phase for orofacial clefts diagnosis, and study the different cleft types in terms of diagnostic methods, timing, and predictability. **Methods:** A search of the PubMed, EMBASE, Scopus, and Web of Science databases was conducted to identify potentially relevant studies published until January 2024. The quality of the selected articles was assessed using the Newcastle–Ottawa scale for methodological quality assessment of cohort studies and the QUADAS-2 scale for diagnostic test studies. **Results:** A total of 18 studies met the eligibility criteria and were included in the review. The findings of this review indicate that the majority of studies showed improved diagnostic accuracy when supplementary techniques, such as 3D ultrasound or magnetic resonance imaging, were added to 2D ultrasound. **Conclusions:** The implementation of magnetic resonance imaging as a standard procedure could significantly improve the precision of diagnosing cleft lip and palate. Therefore, the diagnostic technique used will play a crucial role in the accuracy of the diagnosis.

## 1. Introduction

Orofacial clefts comprise cleft lip (CL) or cleft lip with palate (CLP) and are the most common malformations of the facial area that develop during the sixth–eighth weeks of intrauterine life. They also represent the second most frequent birth defect (13% of all birth defects) [1]. Cleft lip and palate, with a prevalence ranging from 1 in 500 to 1 in 2500 live births and varying by race, occur most frequently among Asians and Native Americans, followed by Europeans, Hispanics, and African Americans [2].

The primary palate includes the lips, mandible, and nasal bone, while the secondary palate includes the hard palate and soft palate. The clefts of the primary and secondary palate have a different embryological origin. Therefore, defects of the secondary palate may or may not include a defect of the soft palate [1].

The severity of the defect depends on the extent of the cleft, ranging from a partial cleft lip to a complete cleft of the lip, jaw, and palate [3]. Clefts can be either unilateral or bilateral and are categorized based on the inclusion of the anterior and/or posterior palate. This condition includes a cleft lip (CL), a cleft palate (CP), or both CL and CP (CLP) [4].

The etiology of these malformations, involving genetic predisposition and fetal exposure to teratogenic factors during the second and early third month, emphasizes that the critical period for cleft development occurs between the fourth and twelfth weeks of intrauterine life [1,2]. Although the etiology is diverse and often uncertain, in the majority of cases, it is associated with a multifactorial condition that involves a combination of genetic and environmental factors [5].

Clefts are associated with numerous factors, but their relevance depends on the timing, location, and phase of morphogenesis, which are crucial for facial development. The principal risk factors include geographical backgrounds intertwined with habits, social circumstances, and access to substances such as drugs, tobacco, and alcohol. The consumption of these substances during pregnancy has been found to strongly correlate with the incidence of and predisposition to hereditary diseases, specifically orofacial cleft [6].

Moreover, diet is a crucial factor associated with orofacial clefts due to its influence on fetal growth during pregnancy. Specifically, a diet rich in folic acid, vitamins, zinc, and other trace elements significantly impacts pregnancy outcomes [6].

Furthermore, certain maternal occupations, such as hairdressing, agriculture, and leather or shoe manufacturing, have been identified as potential contributors to an elevated risk of oral clefts. Additionally, exposure to pesticides, lead, and aliphatic acids has been associated with this increased risk [6]. Therefore, it is imperative to conduct thorough evaluations and examinations of all newborns and their parents, gathering specific data that could prove highly beneficial in both diagnosing and preventing orofacial clefts [6].

Therefore, a precise prenatal diagnosis of cleft lip and palate is essential for establishing comprehensive treatment plans, making accurate prognostic predictions, and providing necessary counseling to parents [2,7].

**Figure 1:**
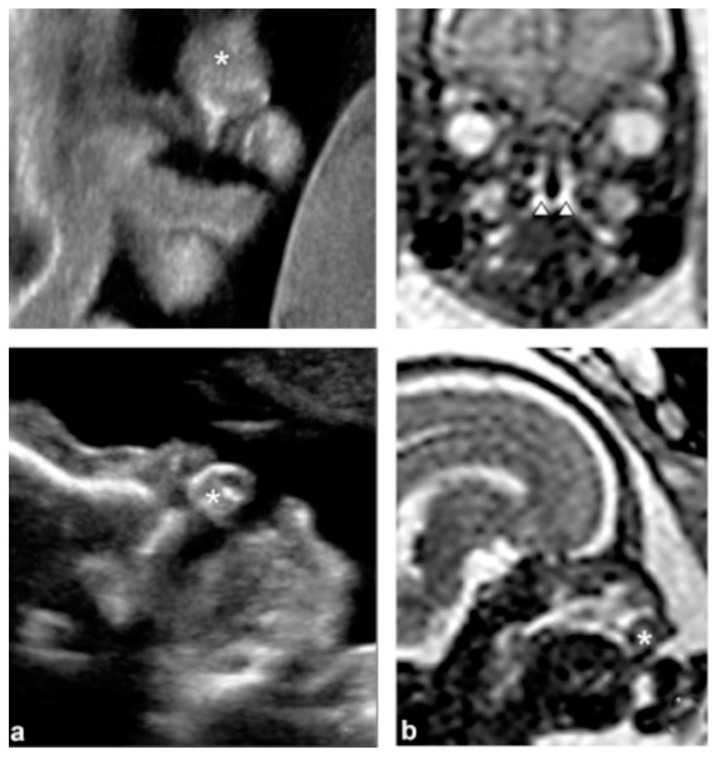
Bilateral cleft lip and alveolus and palate. (**a**) Coronal and sagittal views using 2D ultrasound. * premaxillary protrusion (**b**) Magnetic resonance imaging (MRI) images in the coronal and sagittal planes. * premaxillary protrusion; arrowheads: cleft palate. Reprinted with permission from Ref. [3]. Copyright ® 2016 Elsevier Masson SAS All rights reserved.

Regarding diagnosis methods, the first prenatal ultrasound diagnosis of orofacial cleft was reported in 1981 [8]. Improvements in the resolution of ultrasound equipment and techniques have been made. Accordingly, in 2001, two-dimensional (2D) ultrasound screening became universally used by government guidelines [9], and after 2007, technology underwent extensive development with the availability of three-dimensional (3D) ultrasound [10] (see Figure 1) [3].

However, cleft palate alone is difficult to detect on ultrasound imaging, and the diagnosis of cleft lip with or without cleft palate by ultrasound is not accurate enough in primary care settings [7]. Ultrasound detection results have certain limitations due to factors such as gestational age, amniotic fluid volume, and maternal obesity of pregnant women [11,12]. Consequently, if abnormalities are detected during this 2D ultrasound examination, it is recommended to refer pregnant women with affected fetuses to a tertiary center for a more comprehensive diagnosis [2].

In addition, it is important to note that in utero MRI could be a useful tool for supplementing the diagnosis when fetal ultrasound detects a congenital malformation, especially significant in cases of isolated cleft palates that might not be detected by ultrasound alone [7].

The assertion that prenatal 3D ultrasound and magnetic resonance imaging (MRI) surpass 2D ultrasound in the prenatal diagnosis of orofacial clefts remains to be conclusively established. While certain authors suggest that 3D ultrasonography and MRI might provide enhanced imaging capabilities for detecting posterior clefts [2,3], a comprehensive systematic literature review is necessary to confirm whether these innovative techniques indeed offer advantages in terms of diagnostic accuracy and efficacy.

When orofacial clefts occur, they have a major impact on a baby’s feeding, speech, language and voice formation, breathing, oral functions, bite, tooth formation, and other related issues [6]. Treatment of this anomaly is complex and requires an interdisciplinary team that includes plastic surgeons, otorhinolaryngologists, geneticists, various dental specialists, speech therapists, psychologists, and a coordinating nurse. In addition, it is necessary to monitor these patients for several years until their skeleton reaches full development and maturity [13].

Hence, the primary aim of this study was to perform a systematic review of the accuracy of various imaging techniques in the prenatal diagnosis of cleft lip and palate, specifically 3D ultrasound and/or magnetic resonance imaging alongside 2D ultrasound. Furthermore, this study seeks to evaluate appropriate gestational stages for the identification of orofacial clefts and an in-depth examination of various types of clefts in relation to diagnostic methodologies, timing, and predictability.

## 2. Materials and Methods

The following systematic review was registered in PROSPERO under code CRD42023428743. It was conducted following the guidelines of the PRISMA (Preferred Reporting Items for Systematic Reviews and Meta-Analyses) statement.

### 2.1. PICO Question

The objective was to answer the following PICO (population/patient, intervention, comparison, outcome) question: is the use of 2D ultrasound effective in the diagnosis of prenatal cleft patients compared to the use of 3D ultrasound and/or MRI?

### 2.2. Inclusion and Exclusion Criteria

The study included controlled clinical trials, randomized clinical trials, cohort studies, case–control studies, and diagnostic tests published as ‘Articles’ or ‘Articles in press’. There were no restrictions on the year of publication or language. The inclusion criteria applied were as follows: (1) studies using 2D, 3D ultrasound, and/or MRI to detect cleft lip and palate in fetuses of pregnant women; (2) studies evaluating cleft lip, cleft palate, cleft lip and palate, and cleft lip with alveolar involvement unilateral and/or bilateral; and (3) studies in which the mother and the fetus were evaluated throughout the entire gestation period. Excluded from the study were systematic reviews or meta-analyses, case series, duplicate articles, studies that did not meet the inclusion criteria, and studies that were conducted on prenatal patients with syndromes associated with cleft lip and palate were excluded.

### 2.3. Search Strategy

Detailed search strategies were developed and appropriately revised for each database, considering the differences in controlled vocabulary and syntax rules. The following electronic databases were searched: MEDLINE (Pubmed), EMBASE (Ovid), Web of Science, and Scopus. An electronic gray literature search was conducted using Opengrey. If required, the authors of the articles were contacted by email to request missing information. The reference lists of all eligible studies were also manually searched to identify and screen articles not found in databases that might meet the inclusion criteria. The search was conducted up to January 2024 and sought to identify all articles related to the topic that had been published up to that date. The search attempted to identify all relevant studies regardless of language.

### 2.4. Search Terms

The search strategy utilized a combination of medical subject heading (MESH) terms and free text words for PubMed and was optimized for each database. Boolean operators (“OR” and “AND”) were used to connect terms (MeSH/non-MeSH) relevant to the research question. Searches were conducted for all possible combinations of terms, and the following search equation was used: ((Fetus*) OR (Pregnancy*)) AND ((cleft lip*) OR (cleft palate*)) AND ((ultrasonography*) OR (ultrasonography, prenatal*) OR (prenatal diagnoses*) OR (Magnetic Resonance Imaging*))). All identified articles were exported to Refworks software (ProQuest LLC, Ann Arbor, Michigan, USA) to remove duplicates. The search strategy for all databases is provided in the Supplementary Material (Table S1).

### 2.5. Selection Process

Study selection was performed independently and in duplicate by the first two authors of the review, who were not blinded to the identity of the authors of the studies, their institutions, or the results of their research. The study selection procedure comprised title-reading, abstract-reading, and full-text-reading stages. After excluding ineligible studies, the full report of publications considered eligible for inclusion by either author was obtained and assessed independently. Disagreements were resolved through discussion and consultation with the third author. A record was kept of all decisions regarding study identification, and the reasons for excluding articles were recorded.

### 2.6. Data Extraction and Management

General information was extracted from the selected studies, including authors and year of publication, study design, sample number, type of diagnostic test (2D, 3D ultrasound, and/or MRI), weeks of gestation of the mother, type of orofacial cleft (unilateral/bilateral) and structural involvement (CL, CP, CLP, CL + A), results (n), and article quality according to the Newcastle–Ottawa scale and QUADAS-2.

### 2.7. Quality Assessment

The investigators assessed the quality of the studies independently using the Newcastle–Ottawa scale for cohort studies and the QUADAS-2 scale for diagnostic test studies. Discrepancies between the investigators were resolved by consensus, and a third investigator was consulted in case of doubt.

## 3. Results

### 3.1. Study Selection and Flowchart

**Figure 2:**
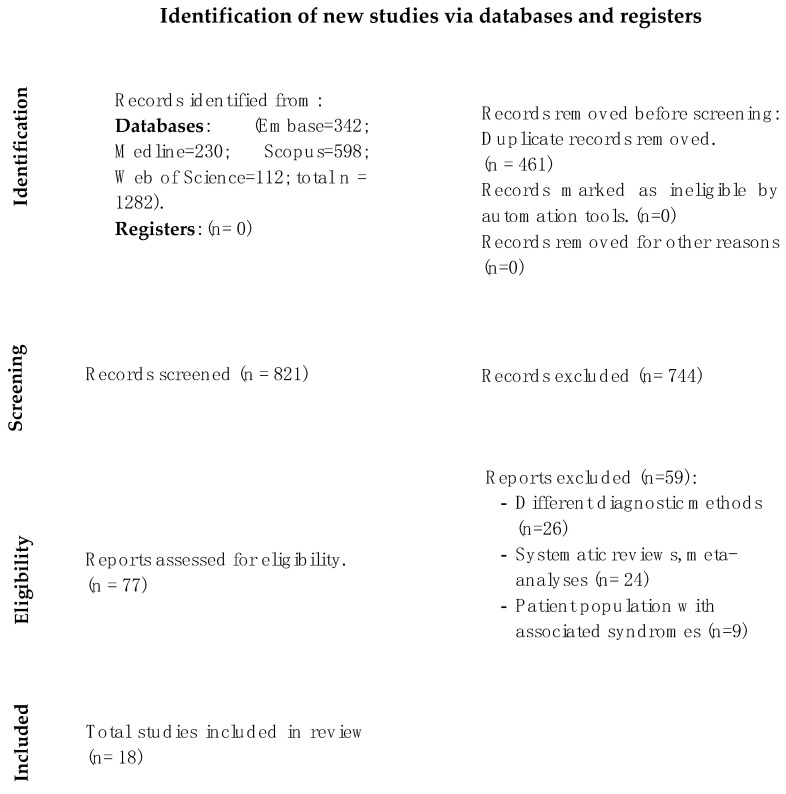
PRISMA 2020 flow diagram for new systematic reviews, which included searches of databases, registers, and other sources.

For the first reading, the title of the article and the abstract of 1282 articles retrieved from the different databases (342 articles from Embase, 230 from PubMed, 598 from Scopus, and 112 from Web of Science) were analyzed. No additional studies were found through manual search. After removing duplicates, the title and abstract were reviewed, and 77 articles were obtained. In the second stage, the full text was read, and the 18 articles that met the criteria were selected. These 18 articles remained for inclusion, and their findings are reported in this systematic review. The PRISMA 2020 flowchart (see Figure 2) provides an overview of the article selection process.

### 3.2. Characteristics of the Included Studies

Table 1 summarizes the characteristics of the 18 studies included in the systematic review. Of these, 7 were diagnostic test studies [3,7,14,15,16,17,18], and 11 were observational studies [19,20,21,22,23,24,25,26,27,28,29]. Of the 11 observational studies (all of them cohort studies), 6 were prospective in design, 4 were retrospective, and only 1 was mixed.

Table 1 shows that out of the 18 articles included in the study, four used only 2D ultrasound and compared it with the gold standard postnatal diagnosis [15,20,21,24], while three studies used 3D ultrasound as a diagnostic test and compared it with the gold standard postnatal definitive diagnosis [14,22,29]. Three studies combined 2D ultrasound with 3D ultrasound to diagnose cleft palate and cleft lip and compared the results with the gold standard postnatal diagnosis [18,26,28]. Additionally, eight studies combined 2D ultrasound with MRI and then compared the results with both the gold standard postnatal diagnosis and with each other [3,16,17,18,23,25,27]. Only singleton pregnancies were evaluated in the included studies.

In terms of the type of sample included in each study, two distinct types can be identified: first, studies in which patients are preselected to undergo the corresponding diagnostic test, resulting in a selection bias, and second, studies in which the diagnostic test is performed on the entire sample and conclusions are drawn by taking into account the diagnosis and comparing it with postnatal findings.

### 3.3. Qualitative Synthesis

#### 3.3.1. Diagnostic Methods and Their Accuracy

Regarding the type of cleft diagnosed by each test in the different studies, the vast majority focus on the visualization of unilateral or bilateral cleft lip (CL), cleft palate alone (CP), and cleft lip with unilateral or bilateral cleft palate (CLP) [3,7,14,15,17,18,21,23,24,25,26,27,28]. Only five articles do not study all of these factors and focus on only one of them [16,19,20,22,29].

Several studies have observed a significant increase in the specificity and sensitivity of diagnostic tests when combining 2D ultrasound with MRI [3,7,16,17,18,23]. For instance, Descamps et al. detected 65.3% correct and 34.7% incorrect orofacial clefts using 2D ultrasound alone but improved their detection to 85.7% correct and 10.2% incorrect orofacial clefts with MRI [16]. Therefore, the positive predictive value and negative predictive value are 96.3% and 80%, respectively. The sensitivity and specificity are 86.7% and 94.1%, respectively [16].

In the study by Shuangshuang et al., a similar trend was observed. However, the sensitivity (89.8%), specificity (99.95%), positive predictive value (95.65%), and negative predictive value (99.88%) exhibited even higher rates [7]. This discrepancy may be attributed to the notably larger sample size in this study (4132 individuals) compared to the previous study (49 individuals).

Nevertheless, in studies using only 3D ultrasound as the diagnostic method, as demonstrated in the study by Ramos et al., the sensitivity and specificity of the test are notably lower and show greater variability. Sensitivity ranges between 33% and 63%, while specificity ranges between 84% and 97% [29].

Finally, it is worth noting that using both 2D and 3D ultrasound together as a diagnostic method leads to varying sensitivity and specificity depending on the type of orofacial cleft observed in the individual, as highlighted in the study by Sommerlad et al. [28]. While the combined use of both tests can improve sensitivity and specificity in detecting cleft lip, effectiveness could be variable in terms of hard and soft palate diagnosis [28].

Therefore, the results of our systematic review indicate that first-trimester ultrasound is a reliable method for diagnosing cleft palates (CPs). The sensitivity of 2D ultrasound alone varies widely, ranging from 43% to 91%, depending on whether cleft lip, isolated cleft palate, or cleft lip and palate (CLP) is being diagnosed. When 2D ultrasound is combined with 3D ultrasound as a diagnostic test, the sensitivity significantly increases, ranging from 84.5% to 100%, and the specificity improves to a range of 84.4–92.8%. Finally, when 2D ultrasound is paired with MRI, the sensitivity remains comparable to the previous scenario, while the specificity notably increases to values between 94.1% and 100% (refer to Table 2 for details).

#### 3.3.2. Phase of Pregnancy at which the Malformation Is Diagnosed

Regarding the timing for the diagnostic test during pregnancy, there is no consensus among the referenced articles. Each study opted for different trimesters to conduct the diagnostic evaluation. The studies conducted by Berggren et al., Baumler et al., Liu et al., Loozen et al., and Maarse et al. agree on conducting the diagnostic test during the second trimester of pregnancy, using either 2D or 3D ultrasound technology [14,15,21,26].

In contrast to the aforementioned studies, Lakshmy et al., Martínez-Diez et al., and Wu et al. investigate the diagnostic reliability of 2D and 3D ultrasound during the first trimester of pregnancy [19,22,24].

Finally, Dabadie et al. and Descamps et al. conduct ultrasound and MRI examinations during the third trimester of pregnancy [3,16], while Shuangshuang et al., Zheng et al., Mailáth-Pokorny et al., Ramos et al., and Sommerlad et al. use ultrasound and/or MRI for prenatal diagnosis of orofacial clefts during the second and third trimesters.

A positive result on second- or third-trimester prenatal fetal US increased the probability of high-risk fetuses having cleft palate from 20% before the test to 92% after the test, while a negative result on second- or third-trimester prenatal fetal US decreased the probability of high-risk fetuses having cleft palate to 3% [30].

When there is suspicion of any orofacial cleft, 3D ultrasound (US) can effectively depict fetal facial anatomy. In the first trimester, utilizing the multiplanar mode display (sagittal, coronal, and axial planes) and 3D surface-rendered reconstruction can successfully visualize clefting of the primary palate and most cases involving the secondary palate more effectively than 2D imaging alone [28,30].

Hence, incorporating MRI during the second or third trimester of gestation would enhance diagnostic accuracy. Similarly, employing 3D ultrasound throughout all three trimesters of pregnancy yields more precise results compared to using 2D ultrasound alone [7,18,27,28,29].

### 3.4. Risk of Bias and Quality Assessment of Individual Studies

The individual parameter scores assessed using the QUADAS-2 scale are illustrated in the Supplementary Materials (Table S2). Among the seven diagnostic test studies, six exhibited a high risk of bias in the selection criteria [3,14,15,16,17,18]. This bias stemmed from non-random sampling, wherein individuals with suspected orofacial clefts were preselected. Only one study conducted the test on all individuals without prior knowledge of whether they had cleft lip and/or cleft palate. In contrast, all studies showed a low risk of bias in other sections.

However, it is important to note the quality of observational articles, specifically cohorts, assessed using the Newcastle–Ottawa scale, which is shown in the Supplementary Material (Table S3). Out of the 11 cohort studies, 4 achieved a score of 8 points [19,20,25,26], 2 scored 9 points [21,24], and 1 scored 7 points [22], indicating that 7 cohort studies had a low risk of bias. In contrast, the four remaining cohort studies scored between 5 and 6 points, indicating moderate-quality studies with a higher risk of bias [27,28,29]. It is important to highlight that in most studies (9 out of 11), the comparability item only scored 1 point. This is because although the different cohorts were comparable with the gold standard, they were not compared with each other.

## 4. Discussion

Although cleft lip is frequently associated with cleft palate, these structural defects differ significantly in their embryological origins and underlying pathophysiology. The primary palate, which includes the upper lip, philtrum, alveolar ridge, and the triangular section of the hard palate in front of the incisor foramen, is the first facial region to develop between the fourth and eighth weeks of gestation. The posterior part of palate development, occurring between the eighth and tenth weeks, is termed the secondary palate and includes the bony segment situated behind the incisive foramen (the hard palate), along with the soft palate or velum [22].

From an embryological perspective, clefts in the secondary palate develop due to a deficiency in the fusion of these palatal processes. At approximately seven weeks of gestation, both palatal processes align vertically alongside the tongue. As development progresses, the tongue moves downward while the palatine processes ascend and merge at the midline, forming the hard palate. This cellular movement also brings together the palatal muscles, culminating in the formation of the muscular segment of the velum [22].

During the initial phase of development, the hard palate widens more rapidly than it lengthens. The process of facial formation is complex, and the small size of these structures presents challenges in diagnosing orofacial clefts during the first trimester of life [22].

Ultrasonography is currently the gold standard for prenatal diagnosis of facial malformations, particularly orofacial clefts. However, the detection results of this method are limited by various factors, such as gestational age, amniotic fluid volume, and maternal obesity. Additionally, certain circumstances, such as oligohydramnios, patient morphology, and late pregnancy, can also affect the accuracy of the results. Furthermore, acoustic shadowing caused by facial bones may hinder proper assessment, particularly of the posterior palate [3].

Several studies have pointed out that MRI plays a positive role in the prenatal diagnosis of fetal cleft lip and palate [3,7,16,17,18,23]. MRI has the advantage of multiple imaging, no ionizing radiation, and relatively objective diagnosis. It is less affected by the clinical experience of the operators, and the fetal position does not significantly impact its visual field.

Therefore, the purpose of this systematic review was to explore the diagnostic value and application of diagnostic methods used in diagnosing fetal cleft lip and palate, aiming to objectively analyze the accuracy of these methods and evaluate the time of gestation at which the malformation is diagnosed.

Regarding studies that use only 2D ultrasound as a diagnostic method, three were conducted during the second trimester of pregnancy [15,20,21]. Additionally, one study aimed to assess the test’s predictive ability in the first trimester of maternal gestation [16]. It is worth noting that larger sample sizes lead to a significant improvement in the test’s predictive capacity. For instance, in a study conducted by Berggren et al. involving 141 patients, the ultrasound’s predictive accuracy was only 31% [15]. In contrast, other studies with sample sizes exceeding 3000 patients have shown predictions ranging from 63% to 75% [15].

Due to the frequent occurrence of clefts affecting the lip and palate, many tertiary care centers have incorporated routine evaluation of the palate. However, assessing isolated clefts of the secondary palate using 2D ultrasound alone presents challenges. Therefore, 3D ultrasound is now being used as an adjunct to aid in diagnosis. Studies have shown that the use of 3D ultrasound significantly improves the sensitivity of diagnosing orofacial clefts that affect both the primary and secondary palate [29].

Studies that exclusively use 3D ultrasound as a diagnostic method note variations in trimester timing. One study was conducted during the first trimester [22], another in the second trimester [19], and the last spanned weeks 12–36 (encompassing the second and third trimesters) [29]. These studies differed notably. The first study involved 240 individuals and explored primary and secondary palate clefts [22], while Baumler et al. and Ramos et al. had smaller sample sizes of 79 and 92 individuals, respectively [14,29]. The former examined all possible cleft types (CL, CP, CLP, and CL + A) [19], whereas the latter focused solely on the secondary palate [29]. As a result, varying outcomes were found. However, an improvement in diagnosing lip and palate clefts was observed when compared to studies that only used 2D ultrasound.

Other studies combined 2D ultrasound with 3D ultrasound [19,26,28]. Lankshmy et al. conducted a study with a smaller sample size (14 patients) but still found that the combination of routine 2D ultrasound with 3D ultrasound in tertiary care centers significantly improved the sensitivity, specificity, and overall accuracy of the test compared to earlier studies [14].

When any type of orofacial cleft is suspected, 3D ultrasound can accurately display fetal facial anatomy compared to 2D. In addition, the type of cleft will also be affected, as highlighted in the study by Sommerlad et al. [28]. While the combined use of both tests may improve sensitivity and specificity in detecting cleft lip, the efficacy may be variable in diagnosing hard and soft palate, as 3D ultrasound can be used to determine whether the defect extends to the alveolar or secondary palate, the type and extension of the fissure, and to show whether the cleft involves the soft palate or hard palate [14,31,32].

The same finding was confirmed by the most recent systematic review published in 2010 regarding this subject, which highlighted the low detection rate of orofacial clefts using 2D ultrasound alone. However, when combined with 3D ultrasound, the detection rates improved significantly [21].

An accurate diagnosis is crucial when advising parents about prenatal findings and preparing them emotionally and practically. Furthermore, determining the specific cleft type is essential in assessing the potential presence of associated severe congenital anomalies, as different subtypes have varying correlations [26].

Although the combined use of 2D and 3D ultrasound improves accuracy in the diagnosis of fetuses with cleft lip and palate, certain limitations must be considered [33]. These include limited resolution, difficulties due to the position of the fetus during ultrasound examination, dependence on operator experience, gestational timing limitations, and soft tissue visualization limitations. Although 3D ultrasound is outstanding in imaging the surface of structures, it can be limited in providing detailed information about the soft tissues within the cleft, which is crucial for comprehensive diagnosis and planning [34]. Patient factors, such as maternal lifestyle, amniotic fluid levels, and the presence of other anatomical abnormalities, can also influence the effectiveness of 3D ultrasound, affecting the quality and interpretation of the images [33,35].

Therefore, fetal MRI stands out as an appealing imaging method for exploring the fetal face in the most recent studies due to its superior resolution, high contrast, multiplanar analysis capabilities, and extensive field of view. Unlike ultrasound, fetal MRI is not hindered by factors such as obesity, late-stage pregnancy, oligohydramnios, or facial bone interference when assessing facial structures. The protocol and MRI sequences are consistent; this makes the anatomical views easier to repeat and more easily understood by surgeons [3].

The last group of articles under consideration involves the integration of routine 2D ultrasound, given to all individuals in each study sample, and MRI, performed only at specialized tertiary care centers for patients with suspected malformations identified in the previous ultrasound tests [3,16,17,18,25,27].

An evident enhancement in the diagnostic accuracy of the test is observed when both imaging techniques are used in combination across all studies. Previously, variations in accuracy, sensitivity, and specificity were noted. However, upon combining 2D ultrasound and magnetic resonance imaging, these values consistently range between 90% and 100%. This robust performance instills confidence and potentially suggests the inclusion of magnetic resonance imaging as a standard technique, as it may have utility for detecting cleft palate in high-risk fetuses.

A recent meta-analysis, comprising eight studies and involving 334 fetuses with a mean gestational age of 27.6 weeks, revealed that MRI exhibits notable diagnostic performance for identifying cleft palate in high-risk fetuses. The findings indicated a pooled sensitivity of 0.97 (95% CI 0.93–0.99), pooled specificity of 0.94 (0.89–0.97), and an area under the curve of 0.98 (95% CI 0.98–0.99). While MRI stands out as the most reliable diagnostic method for cleft palate, its drawbacks include high costs and lengthy wait times, limiting its accessibility primarily to tertiary care centers. In contrast, fetal ultrasound (US) emerges as a preferred choice for second- and third-trimester screening of orofacial clefts due to its accuracy, safety, speed, convenience, and affordability [30].

Considering this outcome, conducting further studies where an MRI is performed on all women, regardless of whether they had a previous risk ultrasound or if they are mothers of children with cleft lip and/or cleft palate, could be beneficial.

This approach could improve the detection of orofacial clefts, increasing the overall sensitivity of prenatal diagnosis and minimizing false negatives. Early counseling in case of prompt detection of clefts could increase parental satisfaction. However, it is crucial to recognize that this screening strategy would increase the overall costs of treatment. Although universal screening by MRI offers advantages in terms of detection and parental satisfaction, its feasibility and cost-effectiveness should be thoroughly evaluated. Before advocating a change in prenatal screening practices, further studies and a thorough analysis of the potential impact on medical outcomes and healthcare costs are essential.

On the other hand, this would significantly increase the cost of treatment [36]. These studies would provide valuable insights into whether MRI is entirely accurate in predicting these conditions. However, the main limitation at present is the cost associated with this test. It involves a significant financial investment, and no studies have been identified where all women in a population undergo routine magnetic resonance imaging during pregnancy.

On the other hand, the potential correlation between the gestational trimester and the efficacy of various tests in detecting cleft palate and/or cleft lip has been examined. Despite the majority of studies performing diagnostic procedures during the second trimester, an analysis of the overall studies indicates no significant correlation between the gestational trimester and the sensitivity, specificity, or diagnostic accuracy [7]. Rather, the accuracy primarily depends on the chosen technique [18,19].

Consequently, the use of MRI improves diagnostic precision regardless of the mother’s gestational week. Similarly, the use of 3D ultrasound combined with 2D ultrasound provides more accurate results than performing 2D ultrasound alone. In addition, 3D ultrasound is a diagnostic technique that is not normally performed as a first option but is performed if a suspected malformation is found on 2D ultrasound.

Finally, the potential for diagnostic enhancements based on the specific type of orofacial cleft has been investigated. Only four studies delved into this aspect, and all of them agree that there is enhanced diagnostic accuracy for cases involving cleft lip or cleft lip together with cleft palate [3,14,17,18]. However, it depends on the technique used [3].

When attempting prenatal diagnoses of cleft alveolar ridge or hard palate, the accuracy decreases, increasing the likelihood of false negative diagnoses [28].

Looking forward, advancements in sonographer proficiency and technology in 2D ultrasound, 3D ultrasound, and MRI are expected to continuously enhance accuracy and detection rates. Due to the relatively lower detection rates of routine 2D ultrasound, it is essential for parents to understand that a negative ultrasound result does not entirely rule out the possibility of their unborn child having an orofacial cleft [26,37]. While 3D ultrasound and MRI can reliably diagnose fetal CL ± P (cleft lip with or without cleft palate), they may not identify cases of CP alone (cleft palate alone) [28]. Hence, it is crucial to refer pregnant women suspected of having an orofacial cleft in the primary care setting to a specialized tertiary care center [38]. Lastly, MRI can provide a more precise depiction of the defect, which can help parents form realistic expectations. The prenatal detection of cleft palate remains a challenge [39]. However, the arrival of artificial intelligence is a positive aspect in addressing these patients, as it has been demonstrated that deep learning models revolutionize the diagnostic process, predict susceptibility to CLP, and enhance alveolar bone grafts and orthodontic treatment [40,41], assisting caregivers in reducing the burden associated with the process of this pathology [42,43].

Limitations within this study arise primarily from its inherent nature, specifically the limited number of eligible studies for inclusion. This study only considered research involving patients prenatally diagnosed with cleft palate and/or cleft lip, including non-syndromic cases, and utilizing 2D, 3D ultrasound, and/or MRI. This limitation is significant because many fetuses with these malformations tend to have associated syndromes, which limits the number of studies that can be included.

Similarly, the differences in methodological designs, search criteria, and quality measurement tools limited the ability to draw conclusions as the collected studies are not fully comparable and a meta-analysis could not be performed.

## 5. Conclusions

While 2D ultrasound is presently the predominant technique for diagnosing orofacial malformations, the incorporation of 3D ultrasound and/or MRI as routine practices could significantly enhance the diagnosis of cleft lip and palate. In contrast to the standard practice of using 2D ultrasound alone, the combination of 2D ultrasound and 3D ultrasound yields superior accuracy. When integrated with MRI, this combined approach exhibits significantly enhanced reliability and specificity.

In addition, it should be noted that most diagnoses occur in the second trimester of pregnancy. Therefore, the accuracy of orofacial cleft detection depends on the gestational week in which the malformation is identified. The choice of diagnostic test is critical in this regard, and although MRI appears to be the most accurate diagnostic test, it is essential to keep in mind that MRI is not feasible during the first trimester of pregnancy.

Furthermore, concerning the specific type of cleft, diagnostic accuracy is higher in cases involving cleft lip and palate. However, when attempting prenatal diagnoses of cleft alveolar ridge or hard palate, the accuracy diminishes, resulting in a higher likelihood of false negative diagnoses.

## Figures and Tables

**Table 1:** Summary of the methodology and results of the studies included in the review.

	Study Design	Number	Type of Diagnostic Test	Weeks of Gestation	Type of Fissure and Structural Involvement	Methodological Quality
Berggren et al., 2012 [15]	Diagnostic test	141	2D US	18–20 weeks	CLP, CL + A CP.	High
Baumler et al., 2011 [14]	Diagnostic test	79	2D and 3D US	2D: 22–25 weeks. 3D: 23–29 weeks.	CL, CP, CLP, CL + A	High
Dabadie et al., 2016 [3]	Diagnostic test	22	2D US and MRI	2D: 24–34 weeks MRI: 27–34 weeks	CL, CP, CLP, CL + A	High
Descamps et al., 2010 [16]	Diagnostic test	49	2D US and MRI	MRI: 24–37 weeks	-	High
Gai et al., 2022 [7]	Diagnostic test	US:110,286 RM: 4132	2D US and MRI	2D: 25 ± 4 weeks MRI: 28 ± 4 weeks	CL, CP, CLP	High
Yan et al., 2022 [17]	Diagnostic test	39	2D US and MRI	-	CL, CP, CLP, CL + A	High
Zheng et al., 2019 [18]	Diagnostic test	88	2D US and MRI	MRI: 19–39 weeks 2D: 19–38 weeks	CL, CLP, CL + A, CP	High
Laifer-Narin et al., 2019 [25]	Cohort study	42	MRI	-	CL, CP, CLP	High
Lakshmy et al., 2017 [19]	Cohort study	US 2D: 2014 US 3D: 14	2D and 3D US	1st trimester	CLP	High
Liu et al., 2017 [20]	Cohort study	3795	2D US	17–18 weeks	CLP	High
Loozen et al., 2015 [26]	Cohort study	134	2D and 3D US	24 weeks + 5 days	CL, CLA, CLP, CP	High
Maarse et al., 2011 [21]	Cohort study	38,760	2D US	18–23 weeks	CL, CLP, CP	High
Mailáth-Pokorny et al., 2010 [27]	Cohort study	34	MRI	14–33 weeks	CL, CLA, CP, CLP	Moderate
Martínez-Diez et al., 2011 [22]	Cohort study	240	3D US	11–13 weeks	CLP	High
Ramos et al., 2010 [29]	Cohort study	92	3D US	12–36 weeks	CP	Moderate
Sommerlad et al., 2010 [28]	Cohort study	124	2D and 3D US	20–34 weeks	CL, cleft alveolar ridge, CP	High
Tian et al., 2019 [23]	Cohort study	71	2D US and MRI	-	CL, CL + A, CLP, CP	High
Wu et al., 2020 [24]	Cohort study	2944	2D US	11–13 weeks	CLP, CP	High

Abbreviations. US: ultrasound; MRI: magnetic resonance; CL: cleft lip; CP: cleft palate; CLP: cleft lip and palate; CL + A: cleft lip with alveolar involvement.

**Table 2:** Summary of accuracy of 2D, 3D, and MRI in diagnosing orofacial clefts.

	Type of Diagnostic Test	Sensitivity	Specificity	Positive Predictive Value	Negative Predictive Value
Berggren et al., 2012 [15]	2D US	43%	100%	-	-
Baumler et al., 2011 [14]	2D and 3D US	100%	90%	97%	100%
Descamps et al., 2010 [16]	2D US and MRI	86.7%	94.1%	96.3%	80%
Gai et al., 2022 [7]	2D US and MRI	81.85%	99.95%	80.14%	99.95%
Laifer-Narin et al., 2019 [25]	MRI	CP: 91.7% CL: 93.1%	-	-	CP: 90% CL: 86.7%
Maarse et al., 2011 [21]	2D US	CL: 81% CLP: 91%	-	-	-
Ramos et al., 2010 [29]	3D US	33–63%	84–97%	-	-
Sommerlad et al., 2010 [28]	2D and 3D US	CL: 95% Cleft alveolar ridge: 84.5% CP: 89.7%	CL: 92.3% Cleft alveolar ridge: 92.8% CP: 84.4%	-	-
Tian et al., 2019 [23]	2D US and MRI	2D US: 77.5% 2D US + MRI: 100%	-	-	-

## Data Availability

Relevant data are contained within the article. Additional data are available from the corresponding author upon reasonable request.

## Supplementary Materials

The following supporting information can be downloaded at: https://www.mdpi.com/article/10.3390/jcm13072090/s1. Table S1: Electronic search strategy for the different databases; Table S2: Quality of observational studies assessed using the QUADAS-2 scale for diagnostic test [3,7,14,15,16,17,18]; Table S3: Quality of observational studies assessed using the Newcastle–Ottawa scale for cohort studies [19,20,21,22,23,24,25,26,27,28,29]; Table S4: PRISMA checklist.
