# Supplementary material for: Diagnostic Methods for the Prenatal Detection of Cleft Lip and Palate: A Systematic Review
Source: J Clin Med. 2024 Apr 3;13(7):2090. doi: 10.3390/jcm13072090 (PMC11012824; doi:10.3390/jcm13072090)
Supplement: Supplementary file 1 [file jcm-13-02090-s001.zip › jcm-2862409-supplementary.pdf]

Diagnostic methods for the prenatal detection of cleft lip and palate. A systematic review.

<u>Online Resource</u>

Table S1 Electronic search strategy for the different databases.

Database	Search strategy
**PUBMED**	*(Fetus[Title/Abstract] OR "Fetal Structures"[Title/Abstract] OR "Fetal Structure"[Title/Abstract] OR "Fetal Tissue"[Title/Abstract] OR "Fetal Tissues"[Title/Abstract] OR Prenatal[Title/Abstract] OR Prenatals[Title/Abstract] OR Antenatal[Title/Abstract] OR Antenatals[Title/Abstract] OR Pregnancy[Title/Abstract] OR Pregnancies[Title/Abstract] OR Gestation[Title/Abstract])*
	**AND**
	("Cleft Lip"[Title/Abstract] OR "Cleft Lips"[Title/Abstract] OR Harelip[Title/Abstract] OR Harelips[Title/Abstract] OR "Cleft Palate"[Title/Abstract] OR "Cleft Palates"[Title/Abstract] OR "Cleft Palate Isolated"[Title/Abstract] OR "Cleft lip and palate"[Title/Abstract] "Cleft lip and palate")
	**AND**
	(Ultrasonography[Title/Abstract] OR "Diagnostic Ultrasound"[Title/Abstract] OR "Diagnostic Ultrasounds"[Title/Abstract] OR "Ultrasound Imaging"[Title/Abstract] OR "Ultrasound Imagings"[Title/Abstract] OR Echotomography[Title/Abstract] OR "Ultrasonic Imaging"[Title/Abstract] OR "Medical Sonography"[Title/Abstract] OR "Ultrasonographic Imaging"[Title/Abstract] OR "Ultrasonographic Imagings"[Title/Abstract] OR Echography[Title/Abstract] OR "Ultrasonic Diagnoses"[Title/Abstract] OR "Ultrasonic Diagnosis"[Title/Abstract] OR "Ultrasonic Tomography"[Title/Abstract] OR "Prenatal Ultrasonic Diagnosis"[Title/Abstract] OR "Prenatal Ultrasonography"[Title/Abstract] OR "Ultrasonic Prenatal Diagnoses"[Title/Abstract] OR "Ultrasonic Prenatal Diagnosis"[Title/Abstract] OR "Prenatal Ultrasonic Diagnoses"[Title/Abstract] OR "Fetal Ultrasonography"[Title/Abstract] OR "Prenatal Diagnoses"[Title/Abstract] OR "Intrauterine Diagnoses"[Title/Abstract] OR "Antenatal Diagnosis"[Title/Abstract] OR "Antenatal Diagnoses"[Title/Abstract] OR "Fetal Screening"[Title/Abstract] OR "Fetal Screenings"[Title/Abstract] OR "Prenatal Screening"[Title/Abstract] OR "Prenatal Screenings"[Title/Abstract] OR "Antenatal Screening"[Title/Abstract] OR "Antenatal Screenings"[Title/Abstract] OR "Fetal Diagnosis"[Title/Abstract] OR "Fetal Diagnoses"[Title/Abstract] OR "Fetal Imaging"[Title/Abstract] OR "Fetal Imagings"[Title/Abstract] OR "Magnetic Resonance Imaging"[Title/Abstract] OR "NMR

	Imaging"[Title/Abstract] OR "MR Tomography"[Title/Abstract] OR "NMR Tomography"[Title/Abstract] OR "Magnetic Resonance Image"[Title/Abstract] OR "Magnetic Resonance Images"[Title/Abstract] OR "MRI Scans"[Title/Abstract] OR "MRI Scan"[Title/Abstract])
	**OR**
	(Fetus[MeSH Terms]) OR (Pregnancy[MeSH Terms])
	**AND**
	(cleft lip[MeSH Terms]) OR (cleft palate[MeSH Terms])
	**AND**
	(ultrasonography[MeSH Terms]) OR (ultrasonography, prenatal[MeSH Terms])) OR (prenatal diagnoses[MeSH Terms])) OR (Magnetic Resonance Imaging[MeSH Terms])
**EMBASE**	*fetus:ti,ab OR 'fetal structures':ti,ab OR 'fetal structure':ti,ab OR 'fetal tissue':ti,ab OR 'fetal tissues':ti,ab OR prenatal:ti,ab OR prenatals:ti,ab OR antenatal:ti,ab OR antenatals:ti,ab OR pregnancy:ti,ab OR pregnancies:ti,ab OR gestation:ti,ab*
	**AND**
	'cleft lip':ti,ab OR 'cleft lips':ti,ab OR harelip:ti,ab OR harelips:ti,ab OR 'cleft palate':ti,ab OR 'cleft palates':ti,ab OR 'cleft palate isolated':ti,ab OR 'cleft lip and palate':ti,ab
	**AND**
	ultrasonography:ti,ab OR 'diagnostic ultrasound':ti,ab OR 'diagnostic ultrasounds':ti,ab OR 'ultrasound imaging':ti,ab OR 'ultrasound imagings':ti,ab OR echotomography:ti,ab OR 'ultrasonic imaging':ti,ab OR 'medical sonography':ti,ab OR 'ultrasonographic imaging':ti,ab OR 'ultrasonographic imagings':ti,ab OR echography:ti,ab OR 'ultrasonic diagnoses':ti,ab OR 'ultrasonic diagnosis':ti,ab OR 'ultrasonic tomography':ti,ab OR 'prenatal ultrasonic diagnosis':ti,ab OR 'prenatal ultrasonography':ti,ab OR 'ultrasonic prenatal diagnoses':ti,ab OR 'ultrasonic prenatal diagnosis':ti,ab OR 'prenatal ultrasonic diagnoses':ti,ab OR 'fetal ultrasonography':ti,ab OR 'prenatal diagnoses':ti,ab OR 'intrauterine diagnoses':ti,ab OR 'antenatal diagnosis':ti,ab OR 'antenatal diagnoses':ti,ab OR 'fetal screening':ti,ab OR 'fetal screenings':ti,ab OR 'prenatal screening':ti,ab OR 'prenatal screenings':ti,ab OR 'antenatal screening':ti,ab OR 'antenatal screenings':ti,ab OR 'fetal diagnosis':ti,ab OR 'fetal diagnoses':ti,ab OR 'fetal imaging':ti,ab OR 'fetal imagings':ti,ab OR 'magnetic resonance imaging':ti,ab OR 'nmr imaging':ti,ab OR 'mr tomography':ti,ab OR 'nmr tomography':ti,ab OR 'magnetic resonance image':ti,ab OR 'magnetic resonance images':ti,ab OR 'mri scans':ti,ab OR 'mri scan':ti,ab
	**OR**
	'fetus'/exp OR 'pregnancy'/exp
	**AND**

	'cleft lip'/exp OR 'cleft palate'/exp
	**AND**
	'ultrasonography'/exp OR 'fetus echography'/exp OR 'magnetic resonance imaging'/exp
**WEB OF SCIENCE**	*TS=(Fetus OR "Fetal Structures" OR "Fetal Structure" OR "Fetal Tissue" OR "Fetal Tissues" OR Prenatal OR Prenatals OR Antenatal OR Antenatals OR Pregnancy OR Pregnancies OR Gestation)*
	**AND**
	TS=("Cleft Lip" OR "Cleft Lips" OR Harelip OR Harelips OR "Cleft Palate" OR "Cleft Palates" OR "Cleft Palate Isolated" OR "Cleft lip and palate")
	**AND**
	TS=(Ultrasonography OR "Diagnostic Ultrasound" OR "Diagnostic Ultrasounds" OR "Ultrasound Imaging" OR "Ultrasound Imagings" OR Echotomography OR "Ultrasonic Imaging" OR "Medical Sonography" OR "Ultrasonographic Imaging" OR "Ultrasonographic Imagings" OR Echography OR "Ultrasonic Diagnoses" OR "Ultrasonic Diagnosis" OR "Ultrasonic Tomography" OR "Prenatal Ultrasonic Diagnosis" OR "Prenatal Ultrasonography" OR "Ultrasonic Prenatal Diagnoses" OR "Ultrasonic Prenatal Diagnosis" OR "Prenatal Ultrasonic Diagnoses" OR "Fetal Ultrasonography" OR "Prenatal Diagnoses" OR "Intrauterine Diagnoses" OR "Antenatal Diagnosis" OR "Antenatal Diagnoses" OR "Fetal Screening" OR "Fetal Screenings" OR "Prenatal Screening" OR "Prenatal Screenings" OR "Antenatal Screening" OR "Antenatal Screenings" OR "Fetal Diagnosis" OR "Fetal Diagnoses" OR "Fetal Imaging" OR "Fetal Imagings" OR "Magnetic Resonance Imaging" OR "NMR Imaging" OR "MR Tomography" OR "NMR Tomography" OR "Magnetic Resonance Image" OR "Magnetic Resonance Images" OR "MRI Scans" OR "MRI Scan")
	AND
	WC=("Dentistry, Oral Surgery & Medicine" OR "Medicine, General & Internal" OR "Obstetrics & Gynecology" OR "Pediatrics" OR "Surgery" OR "Radiology, Nuclear Medicine & Medical Imaging" OR "Remote Sensing")
**SCOPUS**	*(TITLE-ABS-KEY (fetus OR "Fetal Structures" OR "Fetal Structure" OR "Fetal Tissue" OR "Fetal Tissues" OR prenatal OR prenatals OR antenatal OR antenatals OR pregnancy OR pregnancies OR gestation))*
	AND
	(TITLE-ABS-KEY ("Cleft Lip" OR "Cleft Lips" OR harelip OR harelips OR "Cleft Palate" OR "Cleft Palates" OR "Cleft Palate Isolated" OR "Cleft lip and palate"))
	AND
	(TITLE-ABS-KEY (ultrasonography OR "Diagnostic Ultrasound" OR "Diagnostic Ultrasounds" OR "Ultrasound Imaging" OR "Ultrasound Imagings" OR echotomography OR "Ultrasonic

	Imaging" OR "Medical Sonography" OR "Ultrasonographic Imaging" OR "Ultrasonographic Imagings" OR echography OR "Ultrasonic Diagnoses" OR "Ultrasonic Diagnosis" OR "Ultrasonic Tomography" OR "Prenatal Ultrasonic Diagnosis" OR "Prenatal Ultrasonography" OR "Ultrasonic Prenatal Diagnoses" OR "Ultrasonic Prenatal Diagnosis" OR "Prenatal Ultrasonic Diagnoses" OR "Fetal Ultrasonography" OR "Prenatal Diagnoses" OR "Intrauterine Diagnoses" OR "Antenatal Diagnosis" OR "Antenatal Diagnoses" OR "Fetal Screening" OR "Fetal Screenings" OR "Prenatal Screening" OR "Prenatal Screenings" OR "Antenatal Screening" OR "Antenatal Screenings" OR "Fetal Diagnosis" OR "Fetal Diagnoses" OR "Fetal Imaging" OR "Fetal Imagings" OR "Magnetic Resonance Imaging" OR "NMR Imaging" OR "MR Tomography" OR "NMR Tomography" OR "Magnetic Resonance Image" OR "Magnetic Resonance Images" OR "MRI Scans"))

Table S2 Quality of observational studies assessed using the QUADAS-2 scale for diagnostic test.

ARTICLE	SELECCTION	TEST INDEX	REFERENCE TEST	FLOW AND TIME	TOTAL
Berggren et al., 2012 [15]	+	-	-	-	Low probability
Baumler et al., 2011 [14]	+	-	-	-	Low probability
Dabadie et al., 2016 [3]	+	-	-	-	Low probability
Descamps et al., 2010 [16]	+	-	-	-	Low probability
Gai et al., 2022 [7]	-	-	-	-	Low probability
Yan et al., 2022 [17]	+	-	-	-	Low probability
Zheng et al., 2019 [18]	+	-	-	-	Low probability

Abbreviations. “-“: Low probability of bias; “+”: High probability of bias

Table S3 Quality of observational studies assessed using the Newcastle-Ottawa scale for cohort studies.

ARTICLE	SELECTION				COMPARABILITY		EXPOSURE			TOTAL
	1	2	3	4	5a	5b	6	7	8	
Laifer-Narin et al., 2019 [25]	*	*	*	*	*		*	*	*	8/9
Lakshmy et al., 2017 [19]	*	*	*	*	*		*	*	*	8/9
Liu et al., 2017 [20]	*	*	*	*	*		*	*	*	8/9
Loozen et al., 2015 [26]	*	*	*	*	*		*	*	*	8/9
Maarse et al., 2011 [21]	*	*	*	*	*	*	*	*	*	9/9
Mailáth-Pokorny et al., 2010 [27]		*	*		*			*	*	5/9
Martínez-Diez et al., 2011 [22]		*	*	*	*		*	*	*	7/9
Ramos et al., 2010 [29]		*	*		*			*	*	5/9
Sommerlad et al., 2010 [28]	*	*	*		*			*	*	6/9
Tian et al., 2019 [23]	*	*	*		*			*	*	6/9
Wu et al., 2020 [24]	*	*	*	*	*	*	*	*	*	9/9

Table S4. Prisma checklist

Section/topic	#	Checklist item	Reported on page #
**TITLE**			
Title	1	Identify the report as a systematic review, meta-analysis, or both.	1
**ABSTRACT**			
Structured summary	2	Provide a structured summary including, as applicable: background; objectives; data sources; study eligibility criteria, participants, and interventions; study appraisal and synthesis methods; results; limitations; conclusions and implications of key findings; systematic review registration number.	1
**INTRODUCTION**			
Rationale	3	Describe the rationale for the review in the context of what is already known.	1
Objectives	4	Provide an explicit statement of questions being addressed with reference to participants, interventions, comparisons, outcomes, and study design (PICOS).	3
**METHODS**			
Protocol and registration	5	Indicate if a review protocol exists, if and where it can be accessed (e.g., Web address), and, if available, provide registration information including registration number.	3
Eligibility criteria	6	Specify study characteristics (e.g., PICOS, length of follow-up) and report characteristics (e.g., years considered, language, publication status) used as criteria for eligibility, giving rationale.	3
Information sources	7	Describe all information sources (e.g., databases with dates of coverage, contact with study authors to identify additional studies) in the search and date last searched.	3
Search	8	Present full electronic search strategy for at least one database, including any limits used, such that it could be repeated.	4
Study selection	9	State the process for selecting studies (i.e., screening, eligibility, included in systematic review, and, if applicable, included in the meta-analysis).	4
Data collection process	10	Describe method of data extraction from reports (e.g., piloted forms, independently, in duplicate) and any processes for obtaining and confirming data from investigators.	4

Data items	11	List and define all variables for which data were sought (e.g., PICOS, funding sources) and any assumptions and simplifications made.	4
Risk of bias in individual studies	12	Describe methods used for assessing risk of bias of individual studies (including specification of whether this was done at the study or outcome level), and how this information is to be used in any data synthesis.	4
Summary measures	13	State the principal summary measures (e.g., risk ratio, difference in means).	-
Synthesis of results	14	Describe the methods of handling data and combining results of studies, if done, including measures of consistency (e.g., I^2) for each meta-analysis.	-
Risk of bias across studies	15	Specify any assessment of risk of bias that may affect the cumulative evidence (e.g., publication bias, selective reporting within studies).	-
Additional analyses	16	Describe methods of additional analyses (e.g., sensitivity or subgroup analyses, meta-regression), if done, indicating which were pre-specified.	-
**RESULTS**			
Study selection	17	Give numbers of studies screened, assessed for eligibility, and included in the review, with reasons for exclusions at each stage, ideally with a flow diagram.	5
Study characteristics	18	For each study, present characteristics for which data were extracted (e.g., study size, PICOS, follow-up period) and provide the citations.	6
Risk of bias within studies	19	Present data on risk of bias of each study and, if available, any outcome level assessment (see item 12).	9
Results of individual studies	20	For all outcomes considered (benefits or harms), present, for each study: (a) simple summary data for each intervention group (b) effect estimates and confidence intervals, ideally with a forest plot.	8-9
Synthesis of results	21	Present results of each meta-analysis done, including confidence intervals and measures of consistency.	-
Risk of bias across studies	22	Present results of any assessment of risk of bias across studies (see Item 15).	9
Additional analysis	23	Give results of additional analyses, if done (e.g., sensitivity or subgroup analyses, meta-regression [see Item 16]).	6-7
**DISCUSSION**			
Summary of evidence	24	Summarize the main findings including the strength of evidence for each main outcome; consider their relevance to key groups (e.g., healthcare providers, users, and policy makers).	10

Limitations	25	Discuss limitations at study and outcome level (e.g., risk of bias), and at review-level (e.g., incomplete retrieval of identified research, reporting bias).	10
Conclusions	26	Provide a general interpretation of the results in the context of other evidence, and implications for future research.	10
**FUNDING**			
Funding	27	Describe sources of funding for the systematic review and other support (e.g., supply of data); role of funders for the systematic review.	Title page
